# Development of Stored-Product Moths on Cricket Powder and Insect-Enriched Biscuits

**DOI:** 10.3390/foods14183154

**Published:** 2025-09-10

**Authors:** Serena Malabusini, Sara Savoldelli, Andrea Bresciani, Alessandra Marti, Daniela Lupi, Costanza Jucker

**Affiliations:** Department of Food, Environmental and Nutritional Sciences, University of Milan, Via G. Celoria 2, 20133 Milan, Italycostanza.jucker@unimi.it (C.J.)

**Keywords:** *Acheta domesticus*, novel food, Indian meal moth, rice moth, survival, development

## Abstract

In recent years, interest in edible insects has increased in Western countries, leading to an expansion of the market for insect-based products. In this context, it is essential to assess their susceptibility to infestation by stored-product pests, to ensure food safety and to develop appropriate storage management strategies. This study examined the ability of three common stored-product moth species (*Plodia interpunctella, Corcyra cephalonica* and *Ephestia kuehniella*) to infest *Acheta domesticus* powder and biscuits enriched with cricket powder. Larval development, adult emergence, wingspan and female fertility were evaluated. The results showed that *P. interpunctella* and *C. cephalonica* were able to complete their development on cricket powder, albeit with lower survival rates, longer developmental times and fewer offspring than on the standard diet. *E. kuehniella* was unable to develop on cricket powder and only minimal adult emergence was recorded in the biscuit trials, although signs of infestation were detected. These findings demonstrate that stored-product moths represent a potential infestation risk for this novel food, the market for which is expected to grow.

## 1. Introduction

Entomophagy, the practice of eating insects, is common in some areas of the world and at least 2000 insect species have been identified worldwide as edible by humans [1,2]. In Western countries, interest in insects has only increased in recent years, following their inclusion among novel foods under Regulation (EU) 2015/2283, which defines them as “a food or food ingredient that was not used for human consumption to a significant degree within the European Union before 15 May 1997”. The house cricket *Acheta domesticus* (L.) (Orthoptera, Gryllidae) is one of the most promising insects for food because of its nutritional profile [3]. In detail, cricket powder has been found to have a high amount of protein (55–70.75%), fiber (3.5–7%), lipids (9.80–22.80%), ash (3.57–9.10%) and 16.35–22.08% carbohydrates [4,5,6,7,8,9]. In addition, the mineral content in 100 g of the product is as follows: Ca (139–218 mg), K (826–1224 mg), Mg (86–113 mg), Na (263–312 mg), Cu (2.33–4.51 mg), Fe (4.06–5.99 mg), Mn (4.1–12.5 mg) and Zn (12.8–21.8 mg) [7]. However, as highlighted by Meyer-Rochow et al. [10], the chemical composition of the final product can vary considerably depending on the rearing environment, the diet provided, and the processing method applied. Moreover, edible insects may contain anti-nutritional compounds, most commonly oxalates, phytic acid, cyanogenic glycosides, and saponins [11]. To date, only Adeduntan [12] has reported the presence of anti-nutrients (in detail phytate and tannin) in forest crickets; however, no single anti-nutritional compound has yet been identified in *Acheta domesticus*, although it is possible that some may have gone undetected and could pose a health problem in the future [13]. Nevertheless, a study by Gachihi et al. [14] shows that anti-nutrient levels decrease during processing stages, suggesting that appropriate treatment can mitigate potential concerns.

Recently, the European Union authorized *Acheta domesticus* among the insect species approved for human consumption (EU 2035/5—3 January 2023). Its use has been approved both in the form of whole insects or as insect powder; the latter has been used to reformulate food products such as bars, bread, pasta, snacks and other food items [8,15,16]. The use of insect powder allows for greater acceptance by the Western market [8]. In fact, Western consumers may be more likely to try insect-based products rather than whole visible insects [17,18,19,20]. Today, the production and marketing of insect-based foods is growing and, consequently, a major demand for the storage of these products is foreseen. Like any other food ingredient, dried insects could be susceptible to infestation by stored-product pests, which are responsible for millions of euros of lost product each year. It is estimated that post-harvest losses due to insect infestation are 5–10% [21] and can occur during storage, transport, processing, or marketing where stored-product pests find favourable conditions and plenty of food to complete the developmental cycle. Moths of the family Pyralidae are considered to be among the most concerning pests of stored products due to their wide distribution and ability to infest a variety of foods during storage, such as paddy rice and other cereals under high-temperature and -humidity conditions [22]. At present, there is limited knowledge regarding the ability of stored-product pests to infest insect-based products and insect powders. A thorough evaluation of this risk is crucial to better address the pest management strategies for these novel food commodities. Previous studies have focused exclusively on coleopteran species: Rumbos et al. [23] verified that mealworm larvae-meal can be infested by *Tenebrio molitor* L. and *Tribolium confusum* Jaquelin Du Val, while Rigopoulou et al. [24] verified that *Alphitobius diaperinus* (Panzer), *Tenebrio molitor*, *Trogoderma granarium* Everts, *Lasioderma serricorne* F., *Tribolium confusum*, and *Tribolium castaneum* (Herbst) were able to infest pure *A. diaperinus* powder. In another study, the ability to attract the weevil *Sitophilus zeamais* Motschulsky from pasta made with different percentages of cricket powder was assessed, showing the lower attractiveness of the insect-enriched pasta [25]. No information is available for Lepidopteran species that are known to infest amylaceous products rich in carbohydrate like cereal grains [26], flour [27], dried fruits [28,29,30], nuts, seeds, groundnut, cotton, coffee, spices and cocoa beans.

The aim of the current study was to investigate the susceptibility of cricket powder, both in its raw form and as a food ingredient, to infestation by three species of stored-product moths, namely the Indian meal moth, *Plodia interpunctella* (Hübner) (Lepidoptera, Pyralidae), the rice moth, *Corcyra cephalonica* (Stainton) (Lepidoptera, Pyralidae), and the mill moth *Ephestia kuehniella* Zeller (Lepidoptera, Pyralidae). Given that cricket powder can be incorporated into baked goods to enhance their protein content and address the increasing consumer demand for protein-enriched foods, a cricket-enriched biscuit was selected as a model product, owing to its popularity and long shelf life. Specifically, we assessed the ability of the three lepidopterans to complete their life cycle in cricket powder and biscuits and the fertility of females that emerged from them.

## 2. Materials and Methods

### 2.1. Moth Stock Cultures

Stock cultures of *Plodia interpunctella, Corcyra cephalonica* and *Ephestia kuehniella* were set up in Plexiglas cages (60 × 45 × 35 cm) in a climatic room (27 ± 1 °C; 70 ± 5% RH) where the adults could fly and reproduce. The moths had been reared for two years on a standard diet in the laboratory [31]. Adults were captured and placed in a small jar to allow oviposition to collect the eggs. Following [31], eggs were collected and put into Petri dishes (15 cm diameter, 2 cm depth) with a diet used for growing pyralid moths until new adults could emerge.

### 2.2. Description of the Rearing Substrate

As described in Limonta et al. [31], the standard diet (SD) contains 24.5% of bran, 14% of wheat flour, 15% of corn flour, 9% of wheat germ, 6.5% of dry yeast, 14% of honey and 17% of glycerin. The cricket powder (CP) was produced at the DeFENS at University of Milan following Jucker et al. [32]. Crickets were reared under controlled conditions (27 ± 1 °C, 60 ± 5% RH, 12:12 L:D cycle) and were given *ad libitum* access to water and hen feed as food. Seven-to-eight-week-old crickets were collected and, after a 24 h fast, they were rinsed with water, blanched for one minute in boiling water, dehydrated at 50 °C until they reached a constant weight, and ground using a Hawos Queen 2 (Bad Homburg, Germany). The chemical composition of the respective diets used as growth substrates for moths is presented in Table 1.

For the biscuit production, the following formulation was used as a control (W100): wheat flour, water (35 g/100 g flour), sunflower oil (15 g/100 g flour), sugar (15 g/100 g flour), stevia (15 g/100 g flour), chemical yeast (2 g/100 g flour), salt (0.2 g/100 g flour). Cricket-enriched biscuits were prepared by substituting wheat flour with cricket powder and/or hazelnut, as reported in Table 2.

Biscuits were produced by mixing the ingredients in a mixer (KitchenAid, model 5KSM150, Benton Harbor, MI, USA) for two minutes at first speed and another minute at speed two until a smooth dough was obtained. Dough lamination was performed using a mixer tool consisting of rotating rollers with thickness set at 2 mm. Dough was laminated 4 times, obtaining a sheet of 2 mm. Biscuits were then shaped with a cake mold of 7.4 cm length and 4 cm width. Finally, the biscuits were cooked in an air oven (Self Cooking Center; Rational AG, Landsberg Lech, Germany) in static mode for 14 min at 180 °C.

### 2.3. Moth Development and Survival on Cricket Powder and Biscuits

To evaluate the ability of the larvae to survive and complete their development on cricket powder, five replicates were prepared for each lepidopteran species using containers (diameter 10 cm, height 5 cm) filled with 10 g of cricket powder (CP) and five replicates with 10 g of standard diet (SD). For each species, 100 eggs, counted under a stereomicroscope (Leica model MZ9.5, Germany), were placed in each replicate. The three lepidopterans were tested separately.

Similarly, to assess the capability of the larvae to survive and complete development on biscuits with different powder formulations, five replicates for each type of biscuit and each moth species were set up. For each lepidopteran species, 100 eggs were placed in a container (diameter 5.5 cm, height 3 cm) with a mean of 2.84 ± 0.37 g of biscuits.

A total of 30 containers for the powder test, and 60 for the biscuit tests, were prepared for subsequent monitoring. Initially, the containers were sealed with plastic lids to prevent the escape of newly hatched larvae; later, they were replaced with perforated plastic lids covered with wire mesh to ensure adequate aeration and prevent the larvae from escaping. All containers were maintained in a climatic room (27 ± 1 °C; 70 ± 5% RH).

Each replicate was checked three times a week, adding diets and biscuits *ad libitum* to prevent starvation and monitoring the hatching of eggs and the presence of larvae until pupation for up to six months. Adults that emerged in each replicate were counted and placed in the freezer (−20 °C) for further measurements.

### 2.4. Wingspan of Emerged Adults

Collected adults were thawed and sexed using the stereomicroscope by observing the morphological differences in genitalia detectable between males and females [33]. Finally, the wing widths of the specimens that were intact (undamaged or unbroken) were measured using precision calipers (Tesa, Renens, Switzerland) by fixing the insects with entomological pins on polystyrene supports. All data were tabulated for subsequent processing.

### 2.5. Fertility of Females from Cricket Powder Test

To obtain adult individuals from larvae grown on cricket powder and standard diet, Petri dishes (diameter 14.5 cm and height 2 cm) were set up with a random number of eggs from the three lepidopteran species considered. Upon the emergence of new adults, ten pairs (male and female) for each lepidopteran species fed on both diets were formed and placed in containers of 10 cm diameter and 5 cm height. The standard diet served as the oviposition substrate and was chosen to provide a consistent substrate in which to observe the offspring. Adult moths were left in the containers until natural death to ensure completion of female oviposition. All containers were kept in a climatic room. Subsequently, the number of emerged larvae was recorded in each container to assess offspring abundance.

### 2.6. Damage Assessment on Biscuits

In the biscuit tests, signs of larval activity, such as the presence of silky webbing, exuviae or larval excrement, were checked. The number of replicates in which these signs were present was then counted.

### 2.7. Statistical Analysis

The collected data were statistically elaborated using IBM^®^ SPSS^®^ software (Version 24 for Windows, SPSS Inc. Chicago, IL, USA). After checking the homogeneity of the variances through Levene’s test (*p* > 0.05), ANOVA was performed to detect the presence of statistically significant differences between the theses (*p* < 0.05). How diets potentially affected the developmental time (until the emergence of new adults) was analyzed using the statistical software R (version 4.2.0) via parametric cohort survival analyses (Weibull models with a time-dependent hazard function and diet fitted as a factor), with replicates that failed to progress treated as censors [34,35,36].

## 3. Results

### 3.1. Survival and Developmental Time Analysis

**Figure 1:**
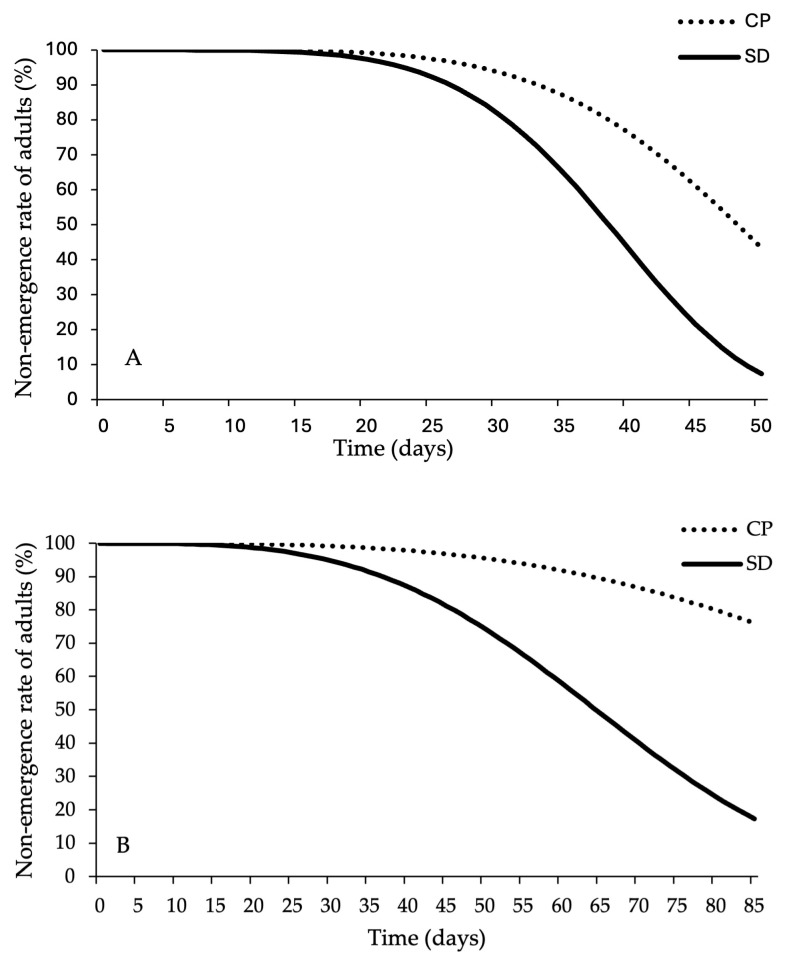
Cohort survival analysis of adult emergence and Weibull model of time from eggs to adult emergence, with replicates where no adults emerged treated as censors. (**A**) *Plodia interpunctella*; (**B**) *Corcyra cephalonica*.

Moth survival, from the egg to adult stage, on cricket powder and standard diet was assessed by counting the number of emerging adults in each replicate. Only *E. kuehniella* did not grow on CP and obtained a mean survival of 69.80 ± 5.74% on SD. Overall, the other two species could survive and develop on CP. For *P. interpunctella*, the mean survival in SD trials was 81.20 ± 3.60%, while on CP 55.20 ± 5.03% (Figure 1A). Similar results were also observed for *C. cephalonica*: the mean survival of individuals in the five replicates was 73.00 ± 2.10% on DS, while on CP this was 24.60 ± 1.99% (Figure 1B).

**Figure 2:**
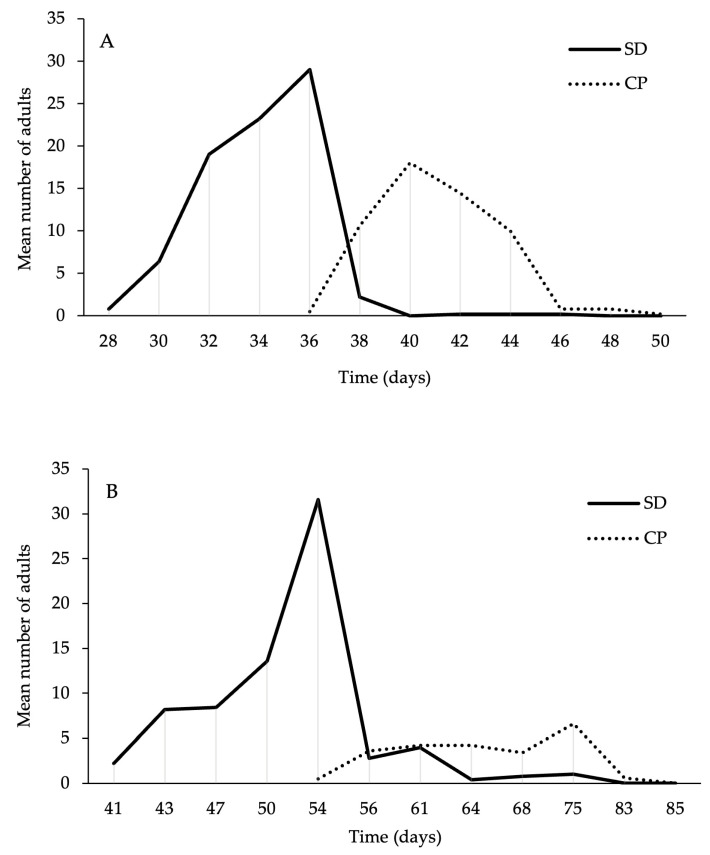
Adult emergence curves of the two moth species analyzed ((**A**) *P. interpunctella*; (**B**) *C. cephalonica*) according to different diets considered.

The duration of development from eggs to adult varied depending on the diet for all moth species, with faster development observed on the SD (cohort survival analysis with censors, *P. interpunctella*: G1 = 209.71, *p* < 0.001; *C. cephalonica*: G1 = 367.11, *p* < 0.001) (Figure 1). Analysis of the adult emergence curves indicated a longer time required for larval development and delayed adult emergence for all tested species when reared on CP. The first adult emergence of *P. interpunctella* on SD was reported 28 days after the start of the trial, compared to 36 days for the emergence of adults on CP. The peak of *P. interpunctella* adults from SD and CP was 36 and 40 days after the start of the trial, respectively (Figure 2A). In the case of *C. cephalonica*, the first adult emergence in the SD tests was observed 41 days after the start of the experimental trial, compared to 54 days required for the first individuals reared on CP. The adult emergence curve (Figure 2B) also indicated that the peak of *C. cephalonica* adult emergence occurred at 54 and 75 days from the start of the trial, respectively, on SD and CP.

Given the low number of adults that emerged on the various biscuit types, the corresponding data on the developmental time are reported exclusively in Table 3. *Ephestia kuehniella* adults did not emerge from any type of biscuit, while adults of the other moths emerged, but in very low numbers. In particular, on the control biscuits (W100), no adults of *P. interpunctella* and *C. cephalonica* emerged, and highest values of survival were observed for *P. interpunctella* on W85C10N5 (1.6%) and W90C10 (1.2%). In all other cases, the emergence rate was less than 1%. The time taken for adult emergence on the biscuits differed in the case of *P. interpunctella* (F_2,15_ = 5.42; *p* = 0.02), with a higher value in W90C10 (137 ± 8 days) and lower in W95N5 (97 ± 10 days). No differences between biscuits were found in the case of *C. cephalonica* (*p* > 0.05) (Table 3).

### 3.2. Wingspan Evaluation

The wingspans of emerged adults from the SD and CP were measured. In all cases and on both substrates, females were significantly larger than males (*P. interpunctella* on SD: F_1,381_ = 572.39; *p* < 0.001; *P. interpunctella* on CP: F_1,275_ = 885.61; *p* < 0.001; *C. cephalonica* on SD: F_1,362_ = 1057.60; *p* < 0.001; *C. cephalonica* on CP: F_1,121_ = 977.35; *p* < 0.001).

Analyzing the wingspan, for both males and females, in *C. cephalonica*, a significantly higher value was found in adults that emerged from larvae reared on SD (males: F_1,224_ = 269.43; *p* < 0.001; females: F_1,259_ = 199.74; *p* < 0.001); on the contrary, this pattern was the opposite in *P. interpunctella* (males: F_1,302_ = 64.71; *p* < 0.001; females: F_1,354_ = 24.87; *p* < 0.001) (Table 4).

Similar results were obtained in biscuit trials, where males, in all cases, being smaller than females (*P. interpunctella* on W85C10N5: F_1,6_ = 33.19, *p* < 0.05; *P. interpunctella* on W90C10: F_1,4_ = 30.5, *p* < 0.05; *P. interpunctella* on W95N5: F_1,2_ = 87.62, *p* < 0.05). Comparing the wingspan with the standard diet of the previous experiment, for both males and females, in almost all cases a significantly higher value was found in adults emerged from larvae reared on SD (*C. cephalonica* females: F_2,204_ = 22.59; *p* < 0.001; *P. interpunctella* males: F_3,159_ = 18.55; *p* < 0.001; *P. interpunctella* females: F_3,234_ = 18.10; *p* < 0.001). This was true except for males of *P. interpunctella*, which were similar to the males that emerged from SD. In detail, the biscuit W85C10N5 obtained significantly bigger wingspasn in both male and female *P. interpunctella* (males: F_2,4_ = 34.71; *p* = 0.003; females: F_2,8_ = 20.85, *p* < 001), and the W95N5 biscuit showed lower values (Table 5).

### 3.3. Fertility of Females from Cricket Powder

**Figure 3:**
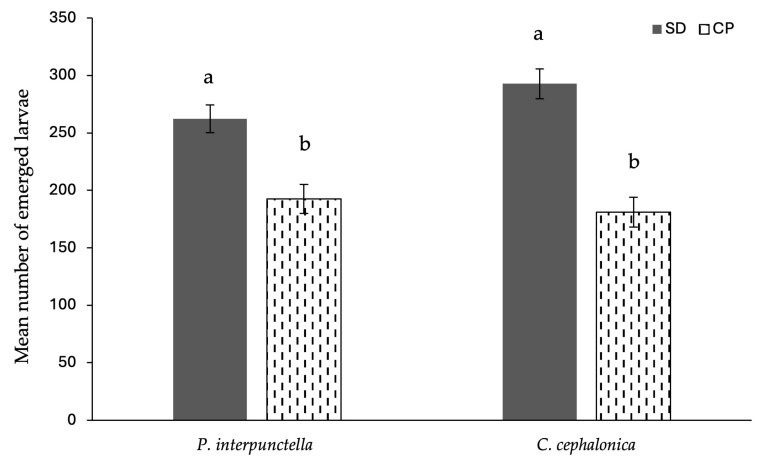
Mean number of emerged larvae (±SE, indicated by error bars) divided by diet and moth species considered. Letters refer to the differences per species between the two diets (*p* < 0.05).

A comparison of new offspring from mating pairs of *C. cephalonica* reared on SD and CP revealed significant differences: the mean number of larvae from SD females was 292.80 ± 13.04, while it was significantly lower for CP females at 180.90 ± 13.04 (F_1,18_ = 36.83; *p* < 0.001) (Figure 3). A similar significant difference was observed in the progeny of *P. interpunctella* females (F_1,17_ = 16.07; *p* < 0.001). One replicate was excluded from the analysis due to the presence of only two larvae, suggesting potential experimental issues (Figure 3). In the biscuit trial, the fertility of the females was not assessed due to the low number of adults that emerged.

### 3.4. Damage Evaluation in Biscuit Trials

The presence of signs of larval activity, such as silk webbing, exuviae or larval excrement, was assessed in trials with the three species, revealing different occurrence percentages. In detail, *P. interpunctella* damaged all biscuits replicates, while *E. kuehniella,* from whose replicates no adults emerged, caused damage only in the first larval stages, but only in 35% of the total replicates, showing a preference for W100 (60%) and W85C10N5 (40%). *Corcyra cephalonica* damaged all replicates, except in the case of W100 (Table 6).

## 4. Discussion

We assessed the susceptibility of cricket powder and biscuits containing different percentages of cricket powder and nuts to three species of stored-product moths, as well as their influence on some biological and morphological traits. The results clearly showed that two of the three lepidopteran species (*P. interpunctella* and *C. cephalonica*) were able to infest *A. domesticus* powder and to complete their development on the tested biscuits. The emergence of new adults was observed in the cricket powder for both species. In contrast, high larval mortality was recorded in the biscuit trials, with less than 2% of individuals successfully emerging as adults.

Although *P. interpunctella* larvae require more protein and fat than *C. cephalonica* [37], it is evident that cricket powder was not optimal for the growth and survival of this moth compared to the standard diet; this is likely due to an inadequate nutritional profile, as similarly observed by Locatelli et al. [38] with other substrates. In fact, compared to the standard diet, all tested species showed lower larval survival and a longer developmental period to reach the adult stage when reared on cricket powder. Damage to the biscuits, assessed by the presence of silk webbing, exuviae, and excrement, was observed across all biscuit types and for all the moth species, including *E. kuehniella*. Despite this evidence of larval activity, which occurred in 80% of the replicates, successful development to the adult stage occurred in less than 2% of the replicates and was limited to *P. interpunctella* and *C. cephalonica*. The prolonged larval development (up to 152 days for *P. interpunctella*) may be attributed to the low moisture content in the biscuits (3–5%), a factor previously reported to affect development in stored-product insects [39,40,41]. Nevertheless, the limited number of adults emerging from W85C10N5 biscuits suggests that diets containing more protein and fat may support higher survival rates and promote larger adult body sizes [42,43,44].

Only *E. kuehniella* did not grow on cricket powder and biscuits, while some infestation signs were visible on biscuits. Kurtulus et al. [45] studied the influence of different diets on some biological parameters of *E. kuehniella*, showing that the addition of yeast in the diet, resulting in a higher percentage of proteins, shortened the development and increased fecundity. However, the maximum percentage of proteins reached in their experiment was 6.4%, which is much lower than that observed in cricket powder (56%). In our trials, an excess of protein did not allow the growth of *E. kuehniella*; in fact, this species requires a diet rich in carbohydrates [46]. *Ephestia kuehniella* completely failed to survive without carbohydrates and perished as small larvae [47]. On the contrary, other authors have also observed that a diet completely devoid of protein delays development and increases pupae mortality [48].

Larval diet can also strongly influence adult traits (e.g., body size) in various insect species, particularly in those that do not feed during the adult stage and rely on reserves accumulated during preimaginal stages, such as moths and certain fly species [45,49,50,51]. Adult size is a well-known trait influencing fecundity and egg production in several Lepidoptera species [52,53,54,55,56]. This relationship is partly explained by mate preferences, as both sexes tend to select larger partners: larger females generally exhibit higher fecundity, and larger males produce more ejaculate and increase mating frequency [57,58,59]. In the present study, male wing width was always smaller than that of females emerged from all tested substrates, confirming findings previously reported by other researchers [60,61,62,63].

Several authors [64,65] have reported that diet influences the body size and wingspan of *C. cephalonica* adults which, in turn, affects their fecundity. In this study, larger *C. cephalonica* adults were observed on the standard diet, and correspondingly higher offspring production was recorded on this diet compared to specimens fed cricket powder. Conversely, *P. interpunctella* adults that emerged from cricket powder were larger than on the standard diet, possibly due to the higher protein content and the species’ ability to adapt to different substrates, reflecting its high polyphagy and ecological plasticity. Females that emerged from cricket powder showed the capability to oviposit and produce offspring, even if the number of offspring was fewer than those that emerged from a standard diet. In the future, it would be interesting to observe the behaviour of subsequent generations raised on cricket powder to assess their potential preference for this substrate. Similar phenomena have already been reported in other species [66,67], where habituation and associative learning have been documented in phytophagous insects [68]. Furthermore, since the concentrations of specific chemical compounds determine the relative preference of females for oviposition on potential hosts or substrates [69,70], this aspect warrants further investigation. In fact, the present study was conducted under no-choice conditions; however, evaluating the attractiveness of these novel food products to adult females in choice tests would provide more relevant insights and contribute to a better understanding of their behaviour under field conditions. Trematerra et al. [25] conducted a choice test with the maize weevil, *Sitophilus zeamais,* and demonstrated that pasta enriched with cricket powder remained attractive, although its attractiveness decreased as the proportion of cricket powder increased, likely due to masking of the host food’s odour.

The present study showed that cricket powder and cricket-enriched biscuits can be infested by stored-product moths, even though the tested species are not always able to complete their development on these substrates. Therefore, preventing measures, such as proper sanitation management, exclusion practices, temperature and humidity control, and selection of appropriate packaging materials, together with specific monitoring strategies, such as the use of pheromone traps, will be essential in warehouses and production facilities.

## 5. Conclusions

Nowadays, only scarce information is available on the capability of stored pests to infest insect-based products and insect powder, and this evaluation is essential to better address the pest management needed to deal with these new food commodities. The results of this study demonstrated that the survival and development from egg to adult of *P. interpunctella* and *C. cephalonica* are possible on cricket powder, although with significantly lower survival rates compared to the standard diet. Conversely, *E. kuehniella* failed to develop on cricket powder, likely due to its specific nutritional requirement for carbohydrates, which are poorly represented in this protein-rich substrate.

The larval developmental time was longer for cricket powder than for the standard diet, probably due to the different nutritional composition and low moisture content, factors that negatively affect development speed and survival. Although damage was observed on biscuits containing cricket powder, very few adults emerged, indicating that this type of product is not conducive to the completion of the insect life cycle.

Adult size was significantly influenced by diet, with species-specific effects observed. *Corcyra cephalonica* adults were larger on the standard diet, which corresponded with higher fertility. In contrast, *P. interpunctella* adults were larger on the cricket powder diet, suggesting nutritional adaptation and high biological plasticity.

Given the expected expansion of the insect-based product sector in the coming years, implementing effective pest management strategies in this sector is of paramount importance. Our findings indicate that cricket powder—especially as is—the market for which is expected to grow in the coming years, can represent a suitable substrate for the growth and development of major stored-product moths, similarly to conventional food products. This highlights the importance of monitoring and controlling these pests throughout the insect product supply chain in order to ensure safe and pest-free food for the consumer. Future research should focus on assessing the susceptibility of these products to infestation by a broader range of stored-product insects, identifying critical control points within the production and storage chain, and developing targeted management approaches to mitigate potential pest threats.

## Figures and Tables

**Table 1:** Chemical composition (%) of standard diet (SD) and cricket powder produced at DeFENS (CP).

Diets	Protein(%)	Lipid(%)	Fiber (%)	Ash(%)	Carbohydrate(%) *
Standard diet (SD)	12.48	3.98	19.41	2.25	61.88
Cricket powder (CP)	56.07	27.45	5.56	4.56	11.92

* The carbohydrate content was estimated by subtracting the average content of ash, fat, fiber and protein from 100%.

**Table 2:** Biscuit formulations.

Biscuit Name	Powder Mixture (%)
W100	100% wheat flour
W95N5	95% wheat flour + 5% hazelnut flour
W85C10N5	85% wheat flour + 10% cricket powder + 5% hazelnut flour
W90C10	90% wheat flour + 10% cricket powder

**Table 3:** Mean developmental time (days) ± SE of *P. interpunctella* and *C. cephalonica* on biscuits.

	*P. interpunctella*	*C. cephalonica*
W85C10N5	110 ± 7 ab	92 ± 11
W90C10	137 ± 8 b	83 ± 11
W95N5	97 ± 70 a	//

Letters refer to the differences per species between different biscuit types (*p* < 0.05). “//” indicates that there is no data available to calculate the value.

**Table 4:** Mean value (±SE) of wingspan (mm) of adults emerged from the two tested diets.

DIET	*P. interpunctella*		*C. cephalonica*	
	M	F	M	F
SD	14.17 ± 0.05 Bb	16.56 ± 0.06 Ab	18.29 ± 0.07 Ba	22.33 ± 0.09 Aa
	(n = 156)	(n = 129)	(n = 162)	(n = 202)
CP	14.79 ± 0.04 Ba	17.09 ± 0.09 Aa	16.15 ± 0.11 Bb	19.80 ± 0.16 Ab
	(n = 148)	(n = 227)	(n = 64)	(n = 59)

Capital letters refer to the differences between male (M) and female (F) of the same species on the same diets and lower-case letters refer to the differences per species between the two diets (*p* < 0.05).

**Table 5:** Mean value (±SE) of wingspan (mm) of adults emerged from the two tested biscuits.

BISCUITS	*P. interpunctella*		*C. cephalonica*	
	M	F	M	F
W85C10N5	13.49 ± 0.2 Ba	14.85 ± 0.16 Aa	15.28	19.36 ± 0.85 a
	(n = 3)	(n = 5)	(n = 1)	(n = 2)
W90C10	11.61 ± 0.44 Bb	14.60 ± 0.31 Aa	//	17.78 ± 0.86 a
	(n = 2)	(n = 4)		(n = 3)
W95N5	11.02 ± 0.11 Bb	12.41 ± 0.11 Ab	//	//
	(n = 2)	(n = 2)		

Capital letters refer to the differences between males (M) and females (F) of the same species on the same biscuit and lower-case letters refer to the differences per species between different biscuits (*p* < 0.05). “//” indicates that there is no data available to calculate the value.

**Table 6:** Percentage of replicates with signs of larval damage on biscuits.

Species	W100	W85C10N5	W90C10	W95N5	Tot.
*P. interpunctella*	100% (5/5)	100% (5/5)	100% (5/5)	100% (5/5)	100% (20/20)
*C. cephalonica*	80% (4/5)	100% (5/5)	100% (5/5)	100% (5/5)	95% (19/20)
*E. kuehniella*	60% (3/5)	40% (2/5)	20% (1/5)	20% (1/5)	35% (7/20)
Tot.	80% (12/15)	80% (12/15)	73.33% (11/15)	73.33% (11/15)	80% (48/60)

## Data Availability

The original contributions presented in this study are included in the article. Further inquiries can be directed to the corresponding authors.
